# Comparison of the Efficacy of Corticosteroid Injection and Dry Needling in Treating Lateral Epicondylitis: An Observational Study

**DOI:** 10.31729/jnma.v63i2091.9241

**Published:** 2025-11-30

**Authors:** Bikash Thapa, Ritesh Sinha, Sailendra Kumar Duwal Shrestha, Adarsha Mahaseth, Satya Priya Shivakotee, Sujan Shrestha, Manoj Nepali, Sumit Yadav

**Affiliations:** 1 Department of Orthopaedics, Nepal Armed Police Force Hospital, Kathmandu, Nepal; 2 Nepalese Army Institute of Health Sciences, Kathmandu, Nepal; 3 Kantipur Dental College Teaching Hospital and Research Center, Kathmandu, Nepal

**Keywords:** *corticosteroid injection*, *dry needling*, *elbow pain*, *lateral epicondylitis*, *patient-rated tennis elbow evaluation*

## Abstract

**Introduction:**

Lateral epicondylitis is a common cause of lateral elbow pain that impairs grip strength and daily activities. While corticosteroid injection offers rapid symptom relief, dry needling is a newer, minimally invasive technique that may enhance tendon healing and provide longer-lasting benefits. This study compared the short- and long-term efficacy of corticosteroid injection and dry needling in improving pain and function in patients with lateral epicondylitis.

**Methods:**

This prospective comparative observational study was conducted in the Department of Orthopedics, Shree Birendra Hospital, Kathmandu, from June 2022 to July 2023 after obtaining ethical approval from the Institutional Review Committee (Registration number: 492). Sixty-two patients aged I860 years with clinically diagnosed lateral epicondylitis were consecutively assigned to receive either corticosteroid injection (n = 31) or dry needling (n = 31). Pain and function were assessed using the Patient-Rated Tennis Elbow Evaluation questionnaire at baseline, 3 weeks, and 3 months. Intergroup comparisons were made using t-tests or Mann-Whitney U tests, and intragroup differences were analyzed with paired t-tests or Wilcoxon signed-rank tests.

**Results:**

Baseline characteristics were comparable. Corticosteroid injection produced greater short-term improvement at 3 weeks (pain 28.23 ± 10.27 vs 41.71 ± 8.93, p < 0.001), while dry needling showed superior outcomes at 3 months (Patient-Rated Tennis Elbow Evaluation 42.97 ± 16.32 vs 50.45 ± 15.33, p = 0.04). Both interventions achieved significant within-group improvement (p < 0.001).

**Conclusions:**

Corticosteroid injection offers faster initial pain relief, but dry needling yields better long-term functional recovery. Both are effective modalities, with dry needling preferred for sustained management of lateral epicondylitis.

## INTRODUCTION

Lateral epicondylitis (LE), or tennis elbow, is a frequent cause of elbow pain, affecting 1-3% of the general population.^1,2^ It is more common among individuals performing repetitive wrist extension and forearm supination.^3^ In South Asia, prevalence is particularly high in vulnerable groups, such as 39.3% among Pakistani housewives,^4^ while in Nepal, a recent OPD-based study (2025) among police personnel reported 25.8%, though representative community data remain unavailable.^5^ Histopathological findings indicate that LE is a degenerative tendinopathy characterized by microtears and angiofibroblastic proliferation rather than a purely inflammatory disorder^2,6^ This paradigm shift has redirected therapy from anti-inflammatory approaches toward tendon-healing strategies. Current management ranges from rest, physiotherapy, and NSAIDs to corticosteroid injections, dry needling, platelet-rich plasma, and surgery in resistant cases.^7^ Corticosteroid injections provide rapid but short-lived relief with frequent recurrences, whereas dry needling demonstrates more durable functional improvement.^8-10^ This study addresses the need for comparative evidence to guide optimal LE management.

## METHODS

This prospective observational study was conducted in the Department of Orthopaedics from June 2022 to July 2023. Ethical approval was obtained from the Institutional Review Committee (IRC) of Nepalese Army Institute of Health Sciences (Registration number: 492), and written informed consent was taken from all participants. Patients aged 18-60 years with a clinical diagnosis of lateral epicondylitis, confirmed by localized tenderness over the lateral epicondyle and positive Cozen’s, Mill’s, and Maudsley’s tests, were included. Exclusion criteria were a history of elbow trauma, inflammatory arthritis, uncontrolled diabetes, prior elbow surgery, previous corticosteroid injection, or any invasive treatment for lateral epicondylitis within three months of enrollment. In patients with bilateral involvement, only the more symptomatic elbow was considered for analysis.

Participants were recruited using a consecutive non-random sampling technique, enrolling all eligible patients presenting during the study period until the desired sample size was reached. The required sample size was calculated using the following formula:

N = [2 × (Z_α_ + Z_β_)^2^ × SD^2^] / D^2^

Where:

*N* = required sample size per group

*Z_α_* =standard normal deviate for the chosen significance level (1.96 for 95% confidence)

*Z_β_* =standard normal deviate corresponding to desired power (0.84 for 80% power)

*SD* =estimated standard deviation of the outcome measure (obtained from a previous study^7^)

*D* = minimum detectable difference between groups (from the same study^7^)

Substituting the values yielded N ≈ 31 per group. Accordingly, 31 participants were assigned to each group consecutively as they presented: one to the corticosteroid injection group and one to the dry needling group.

In the corticosteroid group, patients received a single injection of 1 ml methylprednisolone (40 mg/ml) mixed with 2 ml of 2% lidocaine at the point of maximal tenderness. In the dry needling group, following infiltration with 2 ml of 2% lidocaine, a 23-gauge needle was introduced at the lateral epicondyle region with maximum tenderness and advanced to the periosteum, which was pricked 10-15 times without withdrawal. No ultrasound guidance was used. All procedures were performed by the same orthopaedic surgeon to minimize operator bias. Patients were instructed to avoid strenuous wrist extension for one week and then gradually resume daily activities. Outcomes were assessed at 3 weeks and 3 months using the Patient-Rated Tennis Elbow Evaluation (PRTEE) questionnaire.

Data were analyzed using SPSS version 25.0. Normality was checked using the Shapiro-Wilk test. Independent-samples t-tests or Mann-Whitney U tests were used for between-group comparisons, and paired t-tests or Wilcoxon signed-rank tests for within-group analyses. Results were expressed as mean ± standard deviation (SD) with corresponding 95% confidence intervals (CIs) to indicate precision. A p-value < 0.05 was considered statistically significant.

## RESULTS

At baseline, the two groups were comparable in demographic and clinical characteristics, with no significant differences in age or sex distribution. Although right-hand dominance was more frequent in the corticosteroid group and left-hand dominance in the dry needling group, this difference did not reach statistical significance, confirming that both cohorts were well matched (Table 1).

**Table 1:** Baseline Demographic and Clinical Characteristics (n = 62).

Variable	Corticosteroid (n=31)	Dry Needling (n=31)	Total (n=62)	p-value
Age (yrs), mean ± SD	89.55±8.01	40.89±8.15	89.97±8.02	0.70^1^
Sex, n (%)				0.79^2^
Female	12(38.70%)	11(35.50%)	23(37.10%)	
Male	19(61.30%)	20(64.50%)	39(62.90%)	
Hand dominance, n (%)				0.054^2^
Right	28(90.30%)	22(71.00%)	50(80.60%)	
Left	3(9.70%)	9(29.00%)	12(19.40%)	

1 Independent-samples t-test

2 Pearson’s Chi-square test

**Table 2:** Pain (PRTEE Pain Subscale) Comparison Between Groups (n = 62).

Time Point	Corticosteroid (mean±SD)	Dry Needling (mean±SD)	Mean Difference (95 % CI)	p-value
Baseline	68.87±10.01	68.68±8.51	-4.81(-9.53 to -0.09)	0.046
8 weeks	28.28±10.27	41.71±8.98	-13.48(-18.37 to -8.60)	< 0.001
8 months	22.97±12.66	28.68±10.41	-0.71(-6.60 to 5.18)	0.81

**Figure 1:**
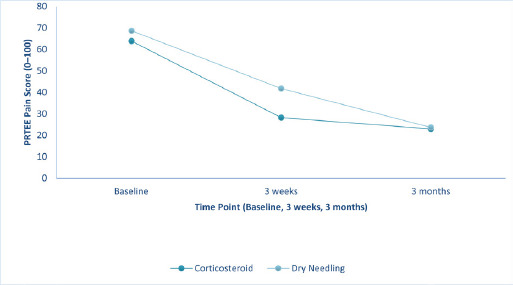
Pain (PRTEE Pain Subscale) over time in corticosteroid and dry needling groups (n=62).

At baseline, pain was slightly higher in the dry needling group. Corticosteroid injection provided significantly greater pain relief at 3 weeks (p < 0.001). Still, by 3 months, both groups showed comparable pain scores (p = 0.81), indicating faster short-term relief with corticosteroid and similar long-term outcomes with dry needling. (Table 2, Figure 1).

**Figure 2:**
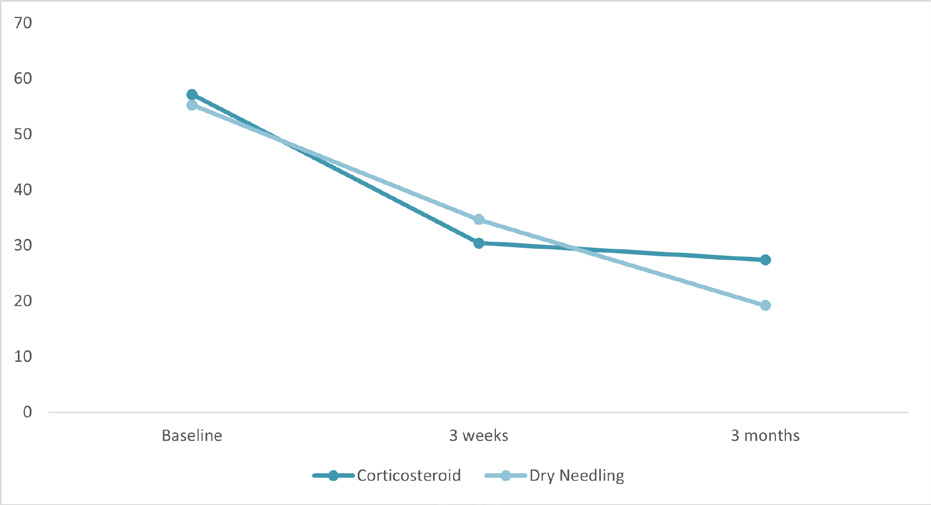
Function (PRTEE Function Subscale) over time in corticosteroid and dry needling groups (n=62).

Baseline functional scores were similar between groups (p = 0.43). At 3 weeks, both showed improvement with no significant difference (p = 0.13). By 3 months, dry needling demonstrated significantly better functional recovery than corticosteroid injection (mean difference = +8.23; p = 0.005) (Table 3, Figure 2).

**Figure 3:**
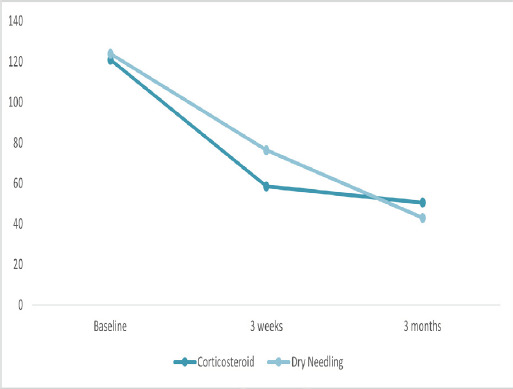
Total PRTEE scores over time in corticosteroid and dry needling groups (n=62).

Baseline total PRTEE scores were comparable between groups (p = 0.38). At 3 weeks, corticosteroid injection showed significantly greater improvement (p < 0.001), indicating better short-term outcomes. By 3 months, dry needling achieved lower (better) PRTEE scores, though the difference was not statistically significant (p = 0.068), suggesting superior long-term trend with dry needling (Table 4, Figure 3).

**Table 3:** Function (PRTEE Function Subscale) Comparison Between Groups (n = 62).

Time Point	Corticosteroid (mean±SD)	Dry Needling (mean±SD)	Mean Difference (95% CI)	p-value
Baseline	57.16±8.40	55.26±10.29	+1.90(-2.87 to 6.67)	0.43
3 weeks	30.45±9.31	34.65±11.97	-4.19(-9.64 to 1.26)	0.13
3 months	27.42±10.66	19.19 ± 11.40	+8.23(2.62 to 13.83)	0.005

**Table 4:** Total PRTEE Score Across Follow-up (n=62).

Time Point	Corticosteroid (mean±SD)	Dry Needling (mean±SD)	Mean Difference (95 % CI)	p-value
Baseline	121.03±11.24	123.97±14.80	-2.94(-9.61 to 3.74)	0.38
3 weeks	58.55±12.35	76.42±16.61	-17.87(-25.31 to -10.44)	< 0.001
3 months	50.45±15.33	42.97±16.32	+7.48(-0.56 to 15.53)	0.068

**Table 5:** Within-Group Clinical Outcomes (Change from Baseline to 3 Weeks and 3 Months) (n=62).

Outcome Measure	Baseline (Mean ± SD)	3 Weeks (Mean ± SD) (95 % CI of Difference)	3 Months Mean ± SD (95 % CI of Difference)	p-value
Corticosteroid Injection				
Pain (PRTEE Pain Subscale)	63.87 ± 10.01	28.23±10.27(Δ=-35.65[-37.99 to -33.30])	22.97±12.66(Δ=-40.90[-43.82 to -37.98])	< 0.001
Function (PRTEE Function Subscale)	57.16 ± 8.40	30.45±9.31(Δ=-26.71 [-28.08 to -25.35])	27.42±10.66(Δ=-29.74 [-31.62 to -27.86])	< 0.001
PRTEE Total Score	121.03 ± 11.24	58.55±12.35(Δ=-62.48 [-65.10 to -59.87])	50.45±15.33(Δ=-70.58 [-73.62 to -67.54])	< 0.001
**Dry Needling**				
Pain (PRTEE Pain Subscale)	68.68 ± 8.51	41.71±8.93(Δ=-26.97 [-28.77 to -25.17])	23.68±10.41(Δ=-45.00 [-46.79 to -43.21])	< 0.001
Function (PRTEE Function Subscale)	55.26 ± 10.29	34.65±11.97(Δ=-20.61 [-22.38 to -18.84])	19.19±11.40(Δ=-36.06 [-37.52 to -34.61])	< 0.001
PRTEE Total Score	123.97 ± 14.80	76.42±16.61(Δ=-47.55 [-50.26 to -44.84])	42.97±16.32(Δ=-81.00 [-83.47 to -78.53])	< 0.001

Note: Δ = mean change from baseline to follow-up with 95 % confidence interval.

Both corticosteroid injection and dry needling produced significant (p < 0.001) improvements in pain, function, and PRTEE scores at 3 weeks and 3 months. In the corticosteroid group, pain decreased from 63.87 ± 10.01 to 28.23 ± 10.27 at 3 weeks (A = -35.65; 95% CI: -37.99 to -33.30) and 22.97 ± 12.66 at 3 months (A = -40.90; 95% CI: -43.82 to -37.98). In the dry needling group, pain reduced from 68.68 ± 8.51 to 41.71 ± 8.93 at 3 weeks (A = -26.97; 95% CI: -28.77 to -25.17) and 23.68 ± 10.41 at 3 months (A = -45.00; 95% CI: -46.79 to -43.21). Overall, corticosteroid injection provided faster early pain relief, while dry needling achieved greater and more sustained improvement by 3 months (Table 5).

All 62 participants completed follow-up with no missing data. Minor transient post-procedural soreness occurred in five patients (8 %), resolving spontaneously within 48 hours; no infections or neurovascular complications were noted.

## DISCUSSION

In our study, the baseline demographic and clinical characteristics between the corticosteroid and dry needling groups were broadly comparable, with no significant differences in age or sex distribution and only a non-significant trend in hand dominance. This finding is consistent with prior randomized and observational studies. Uygur et al., in a prospective randomized trial of 108 patients, similarly reported no significant intergroup differences in age, symptom duration, or baseline PRTEE scores between dry needling and corticosteroid injection groups.^7^ Nagarajan et al. also observed comparable baseline features, noting a nearly identical male predominance of 59-63% across groups.^9^ Wani et al. found similar trends in their 42-patient cohort, with both groups falling within the expected demographic profile of lateral epicondylitis, which most frequently affects adults in their late thirties to mid-forties.^10^

Our sample’s mean age of approximately 40 years aligns with the epidemiologic peak reported in large series, where the highest incidence occurs between 35 and 54 years.^1,2,10^ The male predominance (~63%) also reflects findings from several South Asian cohorts, although some population-based studies have described a more balanced or even female-predominant distribution.^5,11^ For example, Kondaka et al. noted nearequal male-to-female ratios in their observational cohort, underscoring that sex distribution may vary depending on occupational exposures and population sampling.^12^ With respect to hand dominance, our study showed a higher proportion of right-sided involvement overall, with a non-significant trend toward more left-hand dominance in the dry needling group. While previous clinical trials have not consistently stratified outcomes by dominance, epidemiologic data confirm that symptoms most often involve the dominant extremity due to repetitive occupational and sporting activities.^2-5^ These patterns suggest that the minor baseline variation observed in our study is biologically plausible and unlikely to introduce systematic bias.

Our findings regarding pain outcomes are also consistent with existing literature. Corticosteroid injection provided significantly greater short-term pain relief at three weeks compared with dry needling, but by three months, pain scores converged, suggesting comparable long-term control. Similar trends have been reported elsewhere. Uygur et al. found that corticosteroid injection produced faster early pain reduction, whereas dry needling yielded superior benefit at six months.^7^ Nagarajan et al. likewise demonstrated earlier symptom relief with corticosteroid injection, followed by attenuation of differences at later follow-up.^9^ A systematic review by Aman et al. corroborated these findings, concluding that corticosteroid injections are more effective in the short term, while dry needling provides greater durability across tendinopathies, including lateral epicondylitis.^8^ Conversely, Sousa Filho et al. reported that corticosteroid injections may still retain advantages at medium-term follow-up in some musculoskeletal conditions, highlighting heterogeneity in outcomes across trials.^6^ Collectively, these results suggest that corticosteroid injection remains valuable for rapid analgesia, but dry needling represents an equally effective—and in some cases more sustainable—strategy for long-term pain management.

Functional outcomes in our study followed a similar temporal pattern. At baseline, functional scores were comparable between groups, and both interventions produced significant improvements by three weeks. At this early stage, functional recovery was similar, but by three months, dry needling achieved significantly greater improvements, indicating its superiority for sustained functional benefit. This finding is consistent with prior studies. Uygur et al. observed that both groups improved early, but dry needling was associated with greater functional recovery at six months.^7^ Nagarajan et al. likewise reported similar short-term improvements in both groups, with dry needling demonstrating superior recovery at later follow-up.^9^ A systematic review of tendinopathy interventions also concluded that corticosteroid injection provides rapid early gains, whereas dry needling is associated with more durable long-term functional benefit.^8^ Notably, Sousa Filho et al. found that corticosteroid injection sometimes maintained equal or superior function at three months, underscoring variability in the literature.^6^ More recent randomized trials of dry needling for lateral epicondylitis have confirmed functional improvements, though the timing and magnitude of superiority over corticosteroid remain variable.^13^ Taken together, the weight of evidence suggests that corticosteroid injections are effective for early functional recovery, but dry needling offers a more reliable pathway to sustained improvement.

With respect to overall disability, as measured by total PRTEE scores, our study found comparable baseline values between groups but differing patterns of improvement. At three weeks, corticosteroid injection was associated with significantly lower scores, reflecting its well-documented short-term efficacy. By three months, however, dry needling produced significantly greater overall improvement. This mirrors findings from Uygur et al., who demonstrated an early advantage for corticosteroid injection but greater long-term benefit with dry needling.^7^ A systematic review by Aman et al. similarly concluded that corticosteroid injections are superior for early outcomes, while dry needling yields more durable reductions in pain and disability.^8^ In contrast, Sousa Filho et al. reported that some studies favored corticosteroid at medium-term follow-up, and Wani et al. observed corticosteroid superiority at four to eight weeks without the longer-term reversal observed in our study.6,i° Overall, the preponderance of evidence indicates that corticosteroid injection provides rapid, short-term improvements in disability, whereas dry needling offers more sustainable benefit, becoming the preferred option over time.

Finally, our within-group analyses confirmed that both treatments were highly effective in reducing pain and disability relative to baseline. In both groups, pain scores decreased substantially by three weeks and continued to improve by three months, with parallel improvements in functional subscales and total PRTEE scores. These results are consistent with prior randomized controlled trials. Uygur et al. reported significant within-group improvements in both arms at early and late follow-up, confirming the efficacy of each modality.^7^ Similarly, Nagarajan et al. found significant reductions in pain and disability in both groups up to eight weeks.^9^ A recent metaanalysis further supports the within-group effectiveness of dry needling, demonstrating consistent improvements in pain and function across multiple trials.^13^ Systematic reviews have likewise confirmed that both corticosteroid injection and dry needling produce clinically meaningful within-group benefits, though the durability of improvement may vary.^6^ Taken together, this evidence highlights that while between-group differences evolve over time, both interventions are effective in improving outcomes compared to baseline.

In line with our findings, several long-term studies have highlighted concerns regarding the durability of corticosteroid injections and their propensity for symptom recurrence in lateral epicondylitis. Coombes et al. demonstrated in a large randomized controlled trial that although corticosteroid injections achieved rapid early relief, recurrence rates at one year were substantially higher than with placebo (54 % vs 12 %), underscoring their limited long-term efficacy.^14^ Similarly, Bisset et al. reported that nearly three-quarters of initial corticosteroid successes regressed by 52 weeks, whereas physiotherapy maintained superior outcomes beyond six weeks.^15^ A systematic review by Amnan et al. further corroborated these findings, concluding that both corticosteroid and dry needling provide short-term benefit, but dry needling is significantly more effective in sustaining longterm improvement.^8^ Complementing these data, Asghari et al. observed that symptom recurrence following corticosteroid injection typically occurred within six months and was often associated with greater perceived pain and disability than before treatment.^16^ Collectively, these studies reinforce that while corticosteroid injection remains useful for rapid pain relief, regenerative and mechanically stimulatory modalities such as dry needling offer more durable clinical outcomes and lower recurrence risk.

The present study has certain limitations that should be acknowledged. First, it was a non-randomized comparative design, which may introduce selection bias despite comparable baseline characteristics between groups. The relatively small sample size limits the statistical power to detect smaller effect differences, and subgroup analyses could not be reliably performed. Furthermore, the follow-up period of three months was short, preventing assessment of long-term recurrence, tendon healing, or sustained functional outcomes. Future multicentric randomized controlled trials with larger cohorts and extended follow-up are warranted to validate these findings and to evaluate the durability of symptom relief and functional recovery.

Despite these limitations, the findings have practical importance for clinical decision-making in the Nepalese context. Dry needling is a low-cost, minimally invasive procedure that requires only sterile disposable needles and basic training, making it feasible for use in outpatient physiotherapy and orthopedic clinics even in resource-limited settings. Corticosteroid injection, while providing rapid pain relief, incurs recurring costs and requires strict aseptic technique and medical supervision. Given the comparable short-term efficacy but superior long-term outcomes and lower recurrence associated with dry needling, this modality may represent a more sustainable and accessible treatment option for lateral epicondylitis in Nepal, where affordability and follow-up compliance can be challenging. Integration of dry needling into physiotherapy practice, accompanied by patient education and structured rehabilitation, could thus optimize both cost-effectiveness and long-term recovery.

## CONCLUSION

Both corticosteroid injection and dry needling effectively reduced pain and improved function in lateral epicondylitis. Corticosteroid injection provided quicker short-term relief, while dry needling yielded superior long-term recovery. Thus, corticosteroid is suitable for rapid symptom control, whereas dry needling appears preferable for sustained management. Larger randomized trials with longer follow-up are needed to confirm these findings.

## Data Availability

The data are available from the corresponding author upon reasonable request.
